# Development and Characterization of a Caprine Aerosol Infection Model of Melioidosis

**DOI:** 10.1371/journal.pone.0043207

**Published:** 2012-08-15

**Authors:** Carl Soffler, Angela M. Bosco-Lauth, Tawfik A. Aboellail, Angela J. Marolf, Richard A. Bowen

**Affiliations:** 1 Department of Microbiology, Immunology, and Pathology, College of Veterinary Medicine and Biomedical Sciences, Colorado State University, Fort Collins, Colorado, United States of America; 2 Department of Biomedical Sciences, College of Veterinary Medicine and Biomedical Sciences, Colorado State University, Fort Collins, Colorado, United States of America; 3 Department of Environmental and Radiological Health Sciences, College of Veterinary Medicine and Biomedical Sciences, Colorado State University, Fort Collins, Colorado, United States of America; Tulane University School of Medicine, United States of America

## Abstract

Infection with *Burkholderia pseudomallei* causes the disease melioidosis, which often presents as a serious suppurative infection that is typically fatal without intensive treatment and is a significant emerging infectious disease in Southeast Asia. Despite intensive research there is still much that remains unknown about melioidosis pathogenesis. New animal models of melioidosis are needed to examine novel aspects of pathogenesis as well as for the evaluation of novel therapeutics. The objective of the work presented here was to develop a subacute to chronic caprine model of melioidosis and to characterize the progression of disease with respect to clinical presentation, hematology, clinical microbiology, thoracic radiography, and gross and microscopic pathology. Disease was produced in all animals following an intratracheal aerosol of 10^4^ CFU delivered, with variable clinical manifestations indicative of subacute and chronic disease. Bronchointerstitial pneumonia was apparent microscopically by day 2 and radiographically and grossly apparent by day 7 post infection (PI). Early lesions of bronchopneumonia soon progressed to more severe bronchointerstitial pneumonia with pyogranuloma formation. Extrapulmonary dissemination appeared to be a function of pyogranuloma invasion of pulmonary vasculature, which peaked around day 7 PI. Histopathology indicated that leukocytoclastic vasculitis was the central step in dissemination of *B. pseudomallei* from the lungs as well as in the establishment of new lesions. While higher doses of organism in goats can produce acute fatal disease, the dose investigated and resulting disease had many similarities to human melioidosis and may warrant further development to provide a model for the study of both natural and bioterrorism associated disease.

## Introduction

Despite its discovery nearly a century ago [1], melioidosis, or infection with *Burkholderia pseudomallei*, remains an emerging infectious disease of global importance [2]. Infection can occur through percutaneous inoculation, inhalation, or ingestion [3]. Resultant disease can range from an acute, fulminant septicemia to a silent infection that does not result in clinical disease for months to decades later [4]. The clinical signs produced by infection are protean in nature, making diagnosis difficult, especially in non-endemic regions where physicians are unfamiliar with the disease [5].

In northeast Thailand, melioidosis is currently the 3^rd^ leading infectious cause of death, behind human immunodeficiency virus/acquired immunodeficiency syndrome (HIV/AIDS) and tuberculosis [6]. Melioidosis appears likely to displace tuberculosis as the second most common cause of death due to infectious disease if the current trend in melioidosis mortality continues in that locale [6].

Mice have been the predominant host used for studying the pathogenesis and potential therapeutic interventions for melioidosis, and the availability of a large variety of inbred strains, immune function mutants, and immunologic reagents make murine models attractive for the study of this disease [7]. While a great deal has been learned from murine models, the authors are not aware of any papers documenting naturally occurring melioidosis in wild *Mus* spp., despite the very wide host range of animals affected by melioidosis [8]. Wild rodents, specifically rats, were initially believed to be central to the spread of *B. pseudomallei* to humans [9], but subsequent efforts to document disease, carriage, or antibody titers in rats found that infection was exceedingly rare [10], [11]. Given the highly variable nature of human melioidosis, the limited availability of clinical data from murine studies, and the limited publications documenting human pathology/histopathology [12]–[15], it is not possible to conclude that mice are a fully reflective model of naturally-occurring human melioidosis [7].

Goats are naturally affected by melioidosis and offer several potential advantages for use as an animal model of melioidosis, including a large body size allowing clinical monitoring and therapeutic intervention in a manner relevant to humans. Natural disease has been reported to occur in goats from Northern Australia [16]–[20] and Southeast Asia [21]–[24], two highly endemic regions for melioidosis [2], as well as in Aruba [25] and South Africa [26], which have sporadic incidences of disease. In addition to enzootic disease in goats in Northern Australia, outbreaks of melioidosis have also been documented [19], [20]. Both acute and chronic melioidosis occur in goats [8], which encompass a wide variety of clinical signs and pathology [20]. Goats are one of the most frequently affected species [20], [24], and comparatively speaking, there is a good documentation of the clinical signs and pathologic lesions associated with natural disease, which provides a basis of comparison to human disease as well as experimental models.

Two previous experimental models of caprine melioidosis have been published. Narita *et al.* (1982) used a goat isolate of *B. pseudomallei* passaged twice through hamsters prior to intraperitoneal (IP) or subcutaneous (SC) inoculation of goats with either 6.5×10^7^ or 6.5×10^9^ colony forming units (CFU) [27]. Animals infected IP developed septicemic disease of short duration (≤10 d) with microabscesses found throughout the body, while animals infected SC showed no clinical signs other than fever for five days postinfection (PI), but did develop large abscesses in the spleen and lung that were found at necropsy (23 d PI) [27]. Thomas *et al.* (1988) used sheep, goat, or bird isolates of *B. pseudomallei* to infect goats SC with doses ranging from 90 CFU to 5×10^5^ CFU [28]. Goats that received doses greater than or equal to 500 CFU developed fatal disease within two months PI [28]. Goats that received 225 or 90 CFU had signs that varied from no effect to fever and deteriorating condition that necessitated euthanasia [28]. Necropsy revealed abscesses that were most commonly found in the spleen, prescapular lymph node of the inoculated leg, and lungs [28]. Goats without clinical signs had no pathologic lesions when euthanized five months PI [28]. The great disparity between these two experimental models may have been a result of different virulence in the *B. pseudomallei* isolates, as suggested by Thomas *et al*. [28], but was also likely influenced by the different durations of the experiments and the time allowed for disease progression after subcutaneous inoculation.

In the 20 years since description of these original caprine models, which primarily focused on disease in goats and the potential impact on human health from raw milk consumption [28], the research landscape of *B. pseudomallei* and melioidosis has changed considerably. Significant advances have been made in the study of melioidosis, but there is still a great deal about *B. pseudomallei* infection that remains unknown. A greater understanding of pathogenesis and immune function can be achieved through comparative studies of different animal models and human disease. In pursuit of these goals, the objective of this study was to create a non-fulminant, subacute to chronic caprine model of aerosol-transmitted melioidosis. Such disease was readily produced, allowing for the description of the clinical, hematologic, microbiologic, radiographic, and pathologic features of caprine disease, adding a novel and useful model for the study of melioidosis.

## Materials and Methods

### Bacterial Strain and Culture Methods

*B. pseudomallei* (Bp 4176/MSHR 511), isolated from an outbreak at an Australian goat farm [19] was generously provided by Dr. Apichai Tuanyok. Bacteria for infection were grown fresh in Muller-Hinton (MH) broth (M5887 Teknova, Hollister, CA, USA) at 37°C in air with constant shaking at 250 RPM. Bacteria were harvested in mid-log phase growth. Based on the OD600, the culture was diluted in phosphate buffered saline (PBS) to achieve a final concentration of 2×10^4^ CFU/ml. The bacterial suspension used for infection was backtitrated in duplicate on MH agar plates incubated at 37°C in air.

Quantitative blood culture was performed using a pour plate technique modified from Simpson *et al*. [29]. Five milliliters of aseptically collected heparinized blood was mixed with 95 ml of molten (45°C) MH agar and poured into a 15 cm petri dish. Once solidified, the plate was incubated at 37°C in air. Plates were monitored for seven days before being declared negative. Colonies seen on blood culture plates were subcultured onto selective media (MH agar with 4 mg/L gentamicin [MH-gentamicin]) to verify gentamicin resistance and colony morphology consistent with *B. pseudomallei*. Nasal swabs, organ homogenates, and abscess material were also cultured on MH-gentamicin plates at 37°C in air and read at 96 h, 48 h, and 48 h, respectively.

All experiments using *B. pseudomallei* were performed in a biosafety level-3 facility.

### Aerosol Delivery Device

Bacteria were delivered as an intratracheal aerosol. The bacterial suspension was aerosolized using a LC Sprint® nebulizer (mass mean diameter 3.5 µm) and Vios® air compressor (PARI Respiratory Equipment, Inc., Midlothian, VA, USA). The nebulizer was modified so that all exhaled air passed through a HEPA filter. The modified nebulizer was then connected to a cuffed endotracheal tube (size 10) for intratracheal delivery of the aerosol. A target dose of 1×10^4^ CFU in 5 ml of PBS was selected based on pilot studies, which investigated a dose range of 10^4^–10^8^ (data not shown). Nebulization time was approximately 10–15 min. Total dose of bacteria delivered to the lungs was calculated as 10% of the bacteria placed in the nebulizer, a figure that was derived from pilot studies in which two goats were euthanized immediately after exposure and lung tissues samples representing all lung fields were homogenized and plated to determine CFU delivered per gram of lung tissue.

### Experimental Animals

This study was performed in strict accordance with the recommendations in the Guide for the Care and Use of Laboratory Animals of the National Institutes of Health, and every effort was made to minimize suffering. The protocol was approved by the Animal Care and Use Committee of Colorado State University (approval 11-2414A). Group size was determined subjectively based on pilot studies as the study was designed to be descriptive (vs. quantitative) in nature.

Twelve yearling Nubian-cross goats were obtained through private sale for use in this experiment. Goats weighed between 35 to 50 kg. There were seven males and five females. Animals were housed in an ABSL-3 facility for the duration of the experiment. They had *ad libitum* access to water and were fed a complete pelleted feed twice daily. Goats were acclimatized to the facility for approximately one week prior to infection. Infection was performed (Day 0) under intravenous general anesthesia using xylazine hydrochloride (premedication) and ketamine hydrochloride (induction/maintenance). Endotracheal intubation and nebulization was performed with the goats in sternal recumbency.

### Clinical Monitoring: Hematology and Clinical Microbiology

Beginning on Day -4, the goats’ attitude, appetite, and temperature were monitored daily for the duration of the experiment. Normal rectal temperature was defined as 38°C to 40°C. Four pre-infection complete blood counts (CBCs) (HemaTrue Hematology Analyzer, Heska Corporation, Loveland, CO, USA) were performed on each goat (except for goat 26, which had 3) to establish baseline hematologic values. Post-infection CBCs were performed on Days 1–7, 9, 11, 13, 16, 19, and 21.

Nasal swabs and blood cultures were collected on the same schedule as the complete blood counts, except that only one pre-infection sample was collected. For nasal swabs, both nostrils were sampled with a Dacron® fiber tipped plastic applicator swab (Cat. No. 14–959–90, Fisher HealthCare, Houston, TX, USA) and immediately streaked onto a MH-gentamicin agar plate. For blood culture, the collection site over the jugular vein was aseptically prepared with povidone iodine and ethyl alcohol, and 5 ml of venous blood was collected into a heparinized syringe.

### Thoracic Radiography

Three-view thoracic radiographs (right lateral, left lateral, and ventrodorsal) were taken under xylazine sedation for all goats prior to infection and on days 7, 14, and 21. Radiographs of the extirpated lungs were also taken at the time of necropsy. Initial studies showed little to no radiographic change in the lungs on day 2; therefore, this necropsy time point was not aligned with radiography. Radiographs were taken using a MinXRay® 100HF and Agfa® Computed Radiography (CR) 43×35 CR MD 4.0 General Cassettes. During use within the ABSL-3, cassettes were triple sealed in 6 mil polyethelene bags (LAD 8555, Hillas Packaging, Inc., Fort Worth, TX, USA). The cassettes were processed with a CR 85-X digitizer and the images were processed with NX software. All radiographs were read by AJM.

### Euthanasia, Necropsy, Histology, and Organ Burden

Humane euthanasia of three goats with intravenous pentobarbital was planned for days 2, 7, 14, and 21. One goat that was going to be euthanized on Day 2 died during recovery from anesthesia. Necropsies were performed on all goats. Based on organ involvement detected in pilot studies, the following tissues were collected into 10% neutral buffered formalin for histology: mandibular lymph node, retropharyngeal lymph node, tracheobronchial lymph node, mediastinal lymph node, lungs, heart, spleen, liver, kidney, adrenal, thyroid, brain, mesenteric lymph node, and testis or ovary. Samples were routinely embedded in paraffin, sectioned at 5 µm, and stained with hematoxylin and eosin for microscopic evaluation.

In organs with macroscopic abscesses, the abscesses were counted or estimated as a percentage of the total organ volume. Representative abscesses were cultured on MH-gentamicin agar to confirm positive growth of *B. pseudomallei*. In the absence of macroscopic abscesses, samples of the following organs were aseptically collected to calculate the organ burden (CFU/g): lung (cranial, middle, and caudal lobes of left and right lungs), spleen, liver, kidney, retropharyngeal lymph node, tracheobronchial lymph node, and mesenteric lymph node. Tenbroeck homogenizers were used to homogenize 0.5 g of each tissue sample in 2 ml PBS +20% glycerol (limit of detection 50 CFU/g). Neat urine was also collected and cultured from each goat at the time of necropsy. The homogenate or urine (50 µL) was plated in duplicate on MH-gentamicin agar, and the remaining sample was frozen at −80°C. If the colony count was above the limit of quantitation (>300 colonies/plate), the frozen homogenate was thawed and serial log dilutions were plated in duplicate.

## Results

### Bacterial Dose

Infection was performed on four separate days. The backtitration of the nebulized bacterial suspension ranged from 1.07–2.33×10^5^ CFU. The delivered dose to individual goats was approximately one log lower and is reported in Table 1.

**Table 1:** Goats infected with *B. pseudomallei* and status at euthanasia.

Goat No.	Sex	Estimated Dose (CFU) *	Day PI Euthanized	Status at Euthanasia
13	M	1.2×10^4^	2	Febrile, cough present
18	F	1.2×10^4^	2	Febrile
14	M	1.22×10^4^	7	Subclinical disease
19	F	1.2×10^4^	7	Subclinical disease
27	M	2.3×10^4^	7	Febrile
15	M	1.2×10^4^	14	Febrile
20	F	1.2×10^4^	14	Subclinical disease
26	F	1.1×10^4^	14	Afebrile, intermittent cough
16	M	1.2×10^4^	16	Afebrile, moribund, thick nasal discharge
21	F	1.2×10^4^	21	Subclinical disease
22	F	1.2×10^4^	21	Subclinical disease

* Estimated dose delivered to lungs calculated as 10% of the total amount of bacteria placed in the nebulizer (see Methods and Materials).

### Clinical Monitoring

**Figure 1:**
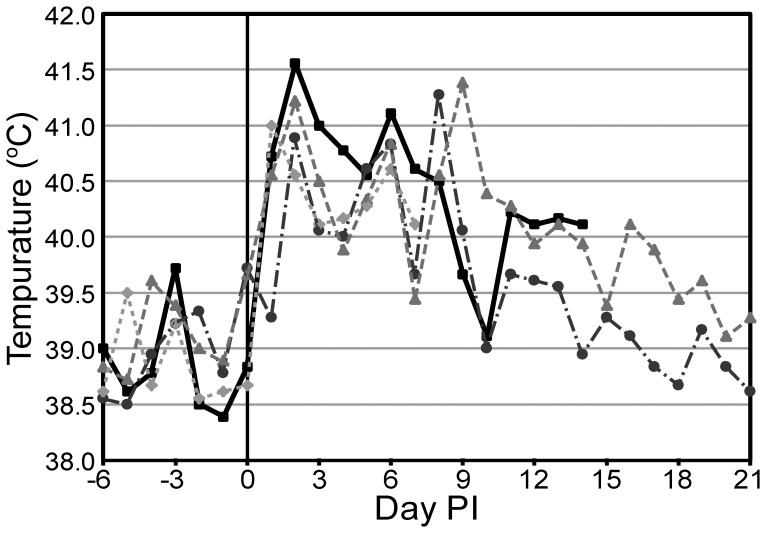
Temperatures from selected goats. Peaks in temperature are seen on days 1 and 2 and between days 6 and 9. Based on the temporospatial distribution of gross lesions, these peaks are likely associated with bacteremic dissemination events even though bacteremia was not detected with blood culture. BpG15 (square), BpG21 (circle), BpG22 (triangle), BpG27 (diamond).

The median rectal temperature for all goats prior to infection was 38.9°C. Ten of 11 goats developed fever by day 1 (median temperature 40.7°C), with the remaining goat developing a fever by day 2 (median temperature 41°C, n = 11). Fevers were variable, but peak temperatures were frequently seen on day 6 (median temperature 40.7°C, n = 9) or day 8 (median temperature 40.9°C, n = 6) after the day 2 peak. Representative temperatures are shown in Figure 1. By day 12 four of six goats had rectal temperatures within the normal range. These four goats all returned to their pre-infection temperatures by the time of euthanasia on days 14 or 21. Despite high fevers (up to 41.6°C), lethargy or decreased appetite was rarely observed.

Other than fever, the most frequent clinical sign recognized was cough, which was observed in seven of 11 goats. Cough was most frequently observed early after infection, days 2–5. Nasal discharge was only observed in Goat 16, which developed on day 11 and persisted until the time of euthanasia on day 16.

### Hematology

Pre-infection hematology revealed a marked lymphocytosis in nearly all goats. The median pre-infection lymphocyte count for all goats over all independent samples was 12.8×10^3^ cells/µL (range 3.5–23×10^3^ cells/µL). This relatively high value was attributed to an epinephrine response, which is often seen in young, excited goats. The median pre-infection granulocyte and monocyte counts for all goats over all independent samples were 5.7×10^3^ cells/µL (range 2.9–15.7×10^3^ cells/µL) and 1.0×10^3^ cells/µL (range 0.4–3.0×10^3^ cells/µL). While some granulocyte and monocyte counts were elevated, no goat maintained a granulocyte or monocyte count above the reference range (1.2–7.2×10^3^ cells/µL and 0.0–1.0×10^3^ cells/µL respectively) for all samples. Variations were attributed to a cortisol response from handling. Pre-infection red cell parameters were generally unremarkable.

**Figure 2:**
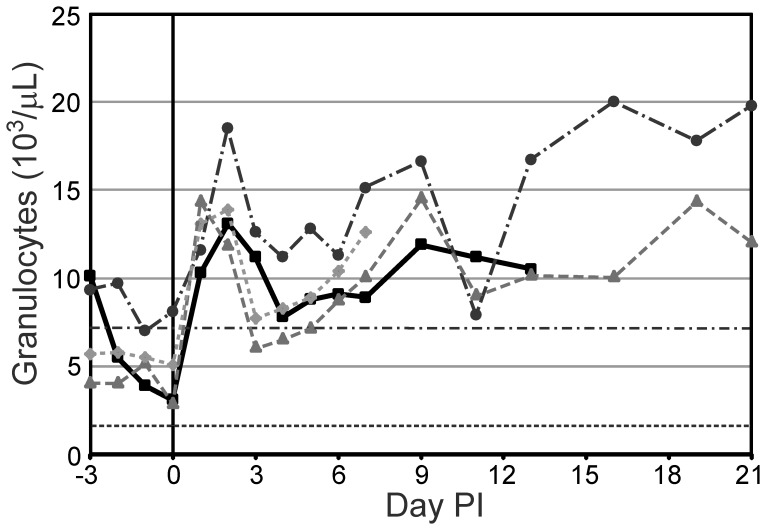
Granulocyte counts from selected goats. During the first half of the study, the granulocyte count appears to mirror the temperature trends seen in Figure 1. BpG15 (square), BpG21 (circle), BpG22 (triangle), BpG27 (diamond), RR High (dash-dot)/Low (dot-dot): reference range limits of normal caprine granulocyte count.

The only cell types to show a marked response post-infection were granulocytes. On day 1 the median granulocyte count increased to 14.2×10^3^ cells/µL and peaked on day 2 at 16.7×10^3^ cells/µL (n = 11). A second peak of 14.9×10^3^ cells/µL was seen on day 9 (n = 6). Representative changes in granulocyte counts can be seen in Figure 2.

### Clinical Microbiology

Nasal swabs were positive for *B. pseudomallei* for 3 of 11 goats on day 1. This was very light growth, with three or fewer colonies per plate. At all other time points, positive nasal swabs were found only for goat 16 on days 5, 11, 13, and 16; growth was light for this goat on days 5 and 11 (less than 20 colonies) and heavy on days 13 and 16 (dense mat of colonies).

Quantitative blood culture was negative for *B. pseudomallei* for all goats except for goat 16, which was positive on day 13, with 1 CFU/5 ml, and on day 16 with 19 CFU/ml.

### Thoracic Radiography

**Figure 3:**
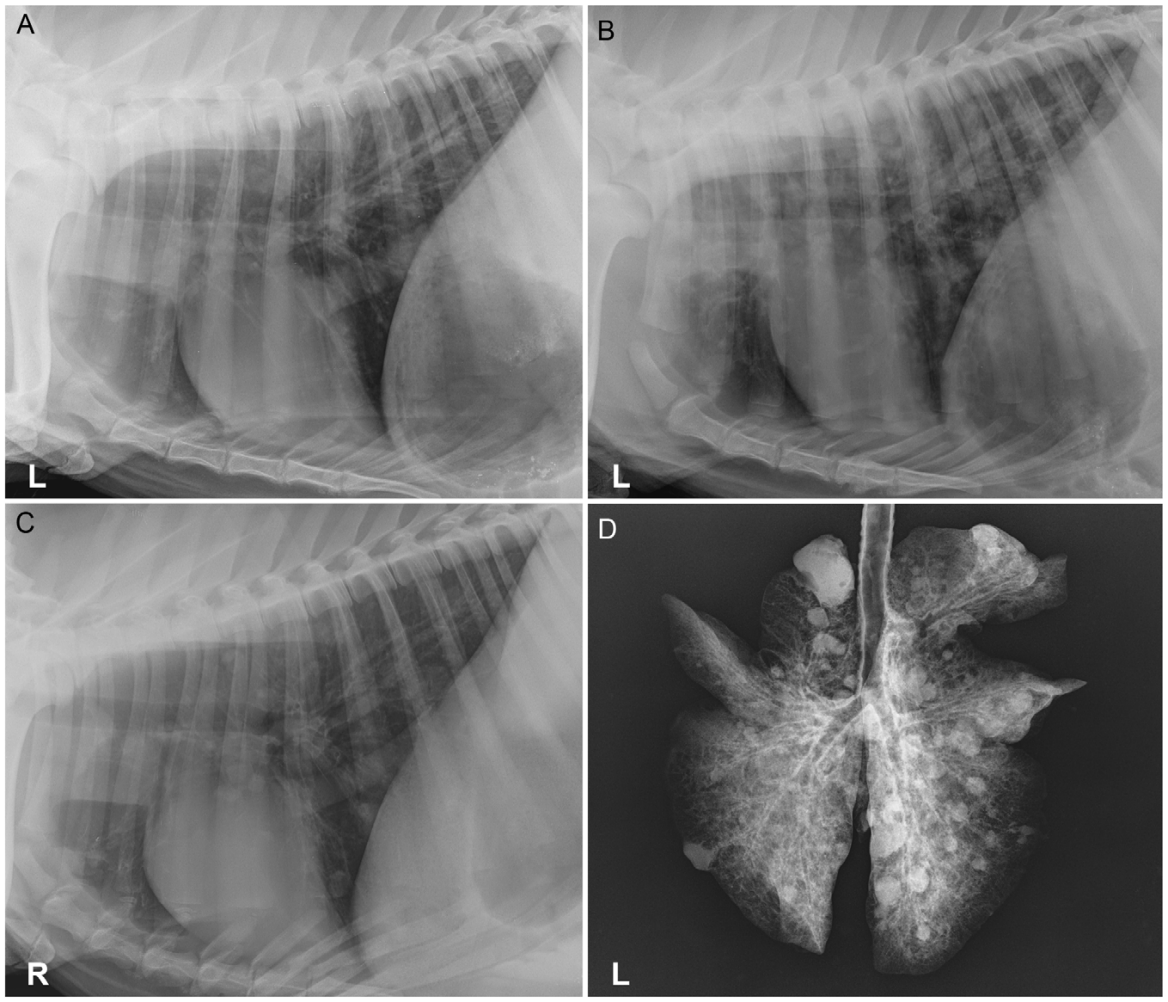
Thoracic and extirpated lung radiographs of goats infected with an intratracheal aerosol of A) Goat 15, day 7, left lateral thorax with a mild bronchointerstitial pattern restricted to the caudal lung lobes. B) Goat 16, day 7, left lateral thorax with moderate to severe bronchointerstitial infiltrates with numerous ill-defined variably sized nodules in all lung lobes with the caudal lung lobes more affected. C) Goat 22, day 21, right lateral thorax with small nodules seen in all lung lobes and mild-moderate bronchointerstitial infiltrates diffusely. D) Goat 22, day 21, extirpated lungs, numerous nodules in all lung lobes, which are much more distinct than in the *in vivo* radiographs.

There were no significant findings on pre-infection thoracic radiographs, except for goat 27, which had a moderate bronchointerstitial pattern; signs of respiratory disease were not evident in this goat prior to infection. Pulmonary disease was radiographically evident in all goats by day 7 (n = 9), which ranged in severity from a mild to moderate bronchointerstitial infiltrates in the caudal lung lobes (Figure 3A) to severe bronchointerstitial infiltrates with variably sized nodules in all lung lobes (Figure 3B). Progressive nodular disease, with an increased number, size, distribution, and/or definition of nodules, was seen in all goats on day 14 (n = 6). Progression of bronchointerstitial infiltrates, with an increase in severity and/or distribution, was seen in four of six goats. The remaining two goats had static bronchointerstitial infiltrates diffusely through all lung lobes. Of the two goats that survived to day 21, both had static, moderate bronchointerstitial infiltrates diffusely. In one goat the nodules had increased in size, but the nodules in the other goat were static. Over the course of infection, the radiographic measurements of the nodules ranged from 4 mm to 27 mm in diameter. When compared to in vivo thoracic radiographs (Figure 3C), radiographs of the extirpated lungs (Figure 3D) were similar, but always showed a greater number of and more clearly defined pulmonary nodules.

### Gross Pathology

Mild focal consolidation in the cranioventral and caudodorsal lung fields was noted in the two goats euthanized on day 2. Retropharyngeal and tracheobronchial lymph nodes were moderately enlarged. No other lesions were noted in any other organs.

**Figure 4:**
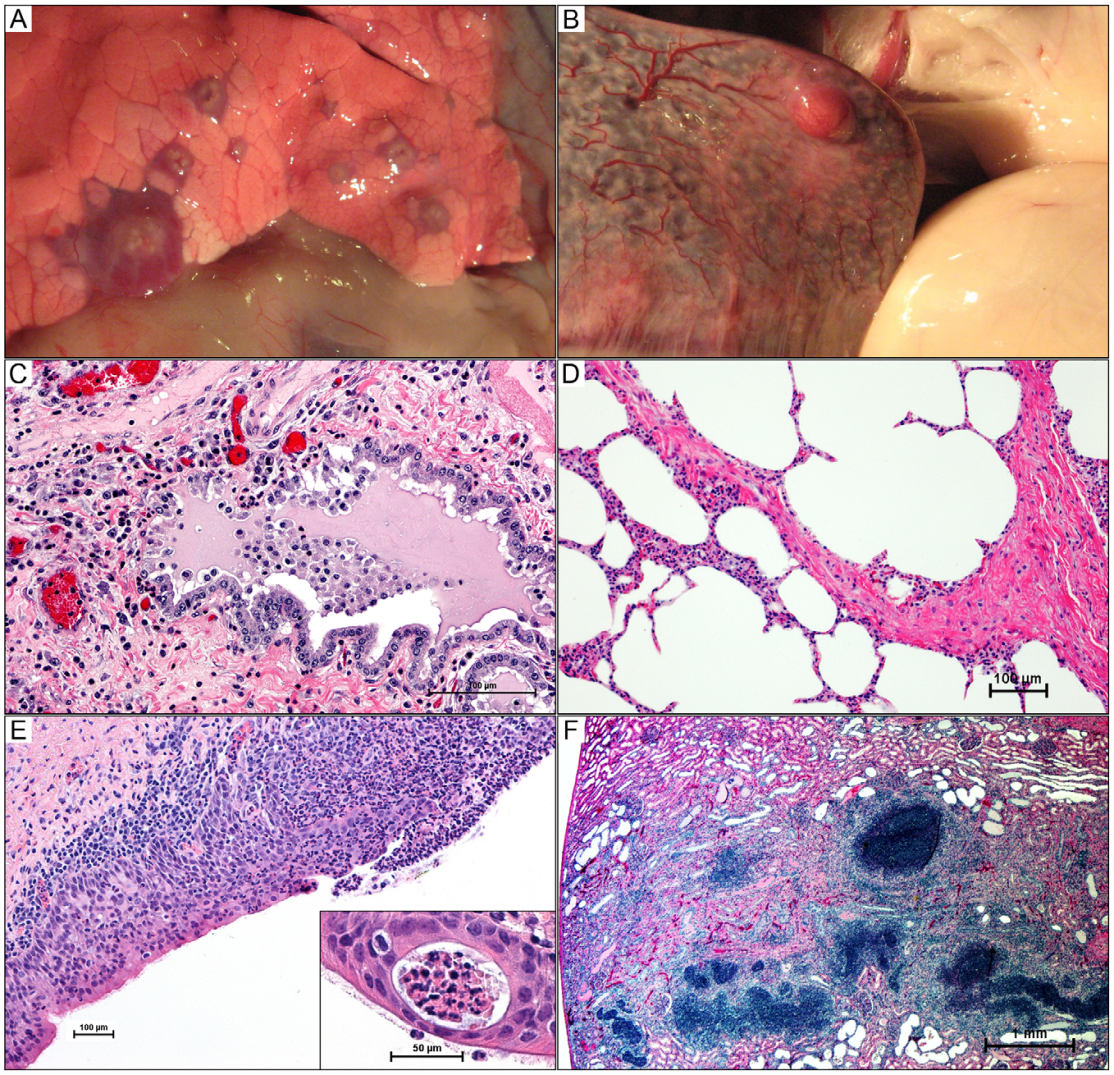
Gross and histologic lesions of caprine melioidosis. A) Lung, multiple discrete subpleural targetoid pyogranulomas with tan purulent centers and consolidated hyperemic rims; B) a splenic subcapsular pyogranuloma bulging over the splenic surface with regional capsulitis and injected vessels; C) bronchiolar epithelium showing apical surface deciliation, segmental necrosis of the pseudostratified ciliated epithelium, neutrophil transcytosis, and luminal aggregates of neutrophils suspended in inflammatory edema; D) pulmonary septal thickening secondary to neutrophil infiltration; E) sever necrosuppurative/ulcerative tracheitis with neutrophil transcytosis and a mucosal abscess (inset); F) linear renal pyogranuloma extending from the cortex down into the medulla obliterating large areas of renal parenchyma. 4C–4F hematoxylin and eosin staining.

By day 7 pulmonary abscessation was present in each of the three goats examined. Abscesses were typically 5 mm or smaller and had a creamy to tan center containing variable amounts of thick purulent material. The parenchyma immediately surrounding the abscess was consolidated, appearing as a rim of dark red tissue around the tan center. This configuration was most easily observed in abscesses just beneath the visceral pleura (Figure 4A). There was individual variation in the absolute number (range 30 to 90) and distribution of abscesses between right and left lungs, but more numerous and larger abscesses were consistently found in the caudal lung lobes. Variable retropharyngeal, tracheobronchial, and/or mediastinal lymph node enlargement was observed in all three goats on day 7, which was typically secondary to lymphoid hyperplasia, but was associated with abscessation of the mediastinal lymph nodes in one goat. A 1 mm abscess was observed in the adrenal gland of another goat after formalin fixation. Gross lesions were not observed in any other organs.

On day 14, pulmonary abscesses remained consistent in appearance and distribution, but varied widely in number (range 12 to 80). The abscesses were larger than at day 7, most were 5–10 mm in diameter, with isolated nodules as large as 20 mm. Retropharyngeal, tracheobronchial, and mediastinal lymphadenomegaly was also consistent with the day 7 findings. Abscessation of the mediastinal lymph node was seen in one goat, which appeared as multiple small (1–2 mm) caseous nodules. This was typical for most lymph node abscesses, which were small in size and multiple in most cases.

Extrapulmonary abscessation was evident in all goats euthanized on day 14. Splenic abscesses (Figure 4B) were seen in all three goats, with one to six abscesses ranging from 2–8 mm in diameter. On cut surface, abscesses were white and appeared solid when small (2–3 mm), but were caseopurulent in appearance when larger (>5 mm). In one goat, a renal abscess was observed beneath the capsule, but not adhered to it. On cut section, the abscess had a linear profile, extending from the cortex down into the medulla, but it did not grossly appear to reach the renal pelvis. The central core of the abscess was white to cream color, which was surrounded by renal parenchyma that was discolored tan.

Goat 16, assigned to the group of three scheduled for euthanasia on day 21, was the most severely affected animal in the study. On day 16, the goat was acutely moribund and was humanely euthanized. Approximately 50% of the lungs were affected with numerous abscesses of varying sizes up to 25 mm in diameter. Abscesses were seen in the retropharyngeal and tracheobronchial lymph nodes, spleen, and kidneys, which were similar in appearance to the abscesses seen at earlier time points. The left adrenal gland was enlarged and edematous, and when cut longitudinally, it was found to contain a solitary abscess that lacked a distinctive structure with no apparent capsule.

Two goats survived with minimal clinically-evident disease until euthanasia on day 21. Pulmonary abscessation with widely disseminated disease was evident in both goats at necropsy. Lung abscesses range from 20 to 45 in number and 10 to 20 mm in diameter. One very large abscess in the cranial lung lobe was adhered the visceral pleura to the rib cage. In both goats, abscesses were seen in the tracheobronchial lymph nodes, spleen (grossly enlarged by >10 abscesses in one goat), and kidney. Abscesses in the mediastinal lymph nodes and one small (2 mm) hepatic abscess were also observed, which were similar in appearance to the small splenic abscesses. Organ involvement for individual goats is summarized in Table 2.

**Table 2:** Summary of organ burdens.

Goat	13	18	14	19	27	15	20	26	16	21	22
*Days PI*	*2*	*2*	*7*	*7*	*7*	*14*	*14*	*14*	*16*	*21*	*21*
Lung	6.5×10^4^	2.4×10^3^	Abs	Abs	Abs	Abs	Abs	Abs	Abs	Abs	Abs
Spleen	NG	NG	NG	NG	NG	Abs	Abs	Abs	Abs	Abs	Abs
Liver	NG	NG	NG	NG	NG	NG	NG	NG	1.2×10^4^	NG	Abs
Kidney	NG	NG	NG	NG	NG	Abs	NG	NG	Abs	Abs	Abs
RPLN	NG	NG	NG	NG	NG	NG	NG	NG	1.7×10^4^	NG	NG
TBLN	8.5×10^2^	NG	6.0×10^2^	2.5×10^2^	NG	6.5×10^2^	NG	NG	Abs	Abs	Abs
MedLN*	NE	NE	NE	NE	Abs	NE	NE	Abs	NE	Abs	NE
MesLN	NG	NG	NG	NG	NG	NG	NG	NG	8.0×10^2^	NG	NG
Urine (cfu/ml)	NG	NG	NG	NG	NG	NG	NG	NG	4.4×10^4^	NG	NG

RPLN: retropharyngeal lymph node, TBLN: tracheobronchial lymph node, MedLN: mediastinal lymph node, MesLN: mesenteric lymph node, NG: no growth of *B. pseudomallei*, NE: not examined, Abs: grossly abscessed

* MedLN only collected if abscessed.

### Bacterial Organ Burden

The bacterial organ burden for all goats is summarized in Table 2. All grossly evident abscesses (or representative abscesses in the case of multiple abscesses within one organ) cultured positive for *B. pseudomallei*. Positive growth was generally restricted to areas of gross lesions, with the exception of day 2 samples and tracheobronchial lymph nodes, both of which often cultured positive in the absence of gross lesions. Multiple organs from goat 16 also cultured positive, with relatively high levels of growth, which appeared to be associated with fulminant septic shock.

Only goat 16 was positive for growth of *B. pseudomallei* from neat urine with 4.4×10^4^ CFU/ml.

### Histopathology

#### Lung

Early bronchiolar lesions at 2 days PI comprised any combination of the following changes: apical surface deciliation; single cell necrosis of the lining pseudostratified ciliated epithelium; neutrophil transcytosis; and luminal aggregates of mucopurulent to fibrinopurulent exudate (Figure 4C). Neutrophilic exudate extended into corresponding respiratory ducts and occasionally into adjacent alveoli with expansion of alveolar septa by variable numbers of macrophages and fewer lymphocytes (Figure 4D). Perivascular edema and small capillary thrombosis with fibrin exudation was also evident in the most affected areas or animals. Some sections presented with prominent lymphangectasia and lymphoid hyperplasia, with many lymphoid follicles having well developed germinal centers.

**Figure 5:**
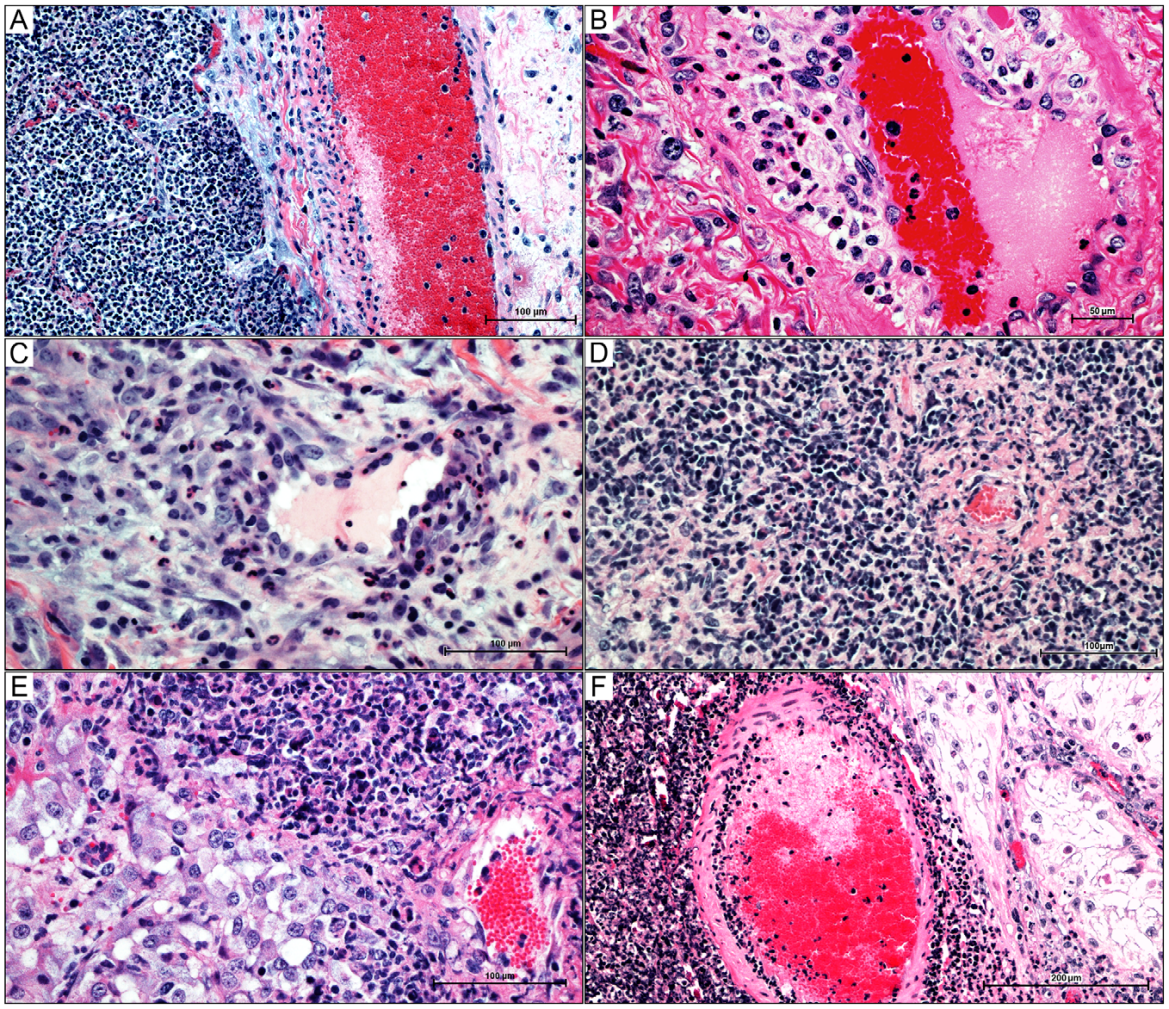
Caprine melioidosis vasculitides in multiple organs. A) pulmonary pyogranuloma encroaching on large interlobular vein, resulting in a segmental leukocytoclastic vasculitis – unaffected endothelium is present on the contralateral side of the vessel; B) pulmonary vasculitis in a medium-sized artery demonstrating a clear separation of the tunica intima from the tunica media by neutrophils and macrophages with segmental intimal proliferation; C) splenic capsular vasculitis with perivascular neutrophilic infiltrate; D) vasocentric renal pyogranuloma showing neutrophil infiltration into the subintima and adventitia with endothelial proliferation and fibrinoid necrosis of a small renal vessel E) focal vasculitis and early pyogranuloma formation at the corticomedullary junction of the adrenal gland; F) leukocytoclastic arteritis in the testicle with an adjacent area of testicular degeneration showing diminished stratification of spermatogenic cells and interstitial suppurative orchitis. 5A–5F, hematoxylin and eosin staining.

By day 7 well-formed abscesses were cuffed by mantles of macrophages (including epithelioid histiocytes) and lymphocytes, which in turn were ensheathed by fibrous connective tissue capsules of varying thickness with a significant histiocytic component. Many of the intervening alveolar spaces were lined by plump cuboidal epithelium indicative of proliferation of type 2 pneumocytes. Interlobular septa were also thickened by edema and inflammatory infiltrates, extending to the pleura. Pleural surfaces were variably expanded by pleocellular exudate, subpleural edema, fibroplasia, and lymphangectasia and lymphangitis. A prominent segmental leukocytoclastic vasculitis with perivascular cuffs of lymphocytes and histiocytes (Figure 5A and 5B) primarily effecting veins was also present.

Lesions were less exudative and more chronic on day 14, with multiple coalescing pyogranulomas and suppurative to fibrinopurulent bronchopneumonia. Goat 16, which developed terminal disease on day 16, showed marked pulmonary pathology. Vascular thromboses with perivascular hemorrhages were evident throughout the lungs, which showed fibrin exudation into parenchyma and interlobular septa, prominent peribronchiolar lymphoid hyperplasia, and chronic pleuritis with lymphangectasia and lymphangitis. Lesions on day 21 were more of chronic bronchointerstitial pneumonia with septal fibrosis, multifocal coalescing pyogranulomas, multifocal atelectasis, and pleural fibrosis.

#### Trachea

Variable tracheitis, with mucous gland hyperplasia, neutrophil transcytosis, and mucosal abscesses (Figure 4E), were seen in all goats at all time points. The severity of the suppurative process appeared most intense on day 2, and then seemed to decrease in severity with time, having only a mild, focal tracheitis present on day 21. The exception to this pattern was goat 16 that developed terminal disease, which had severe necrosuppurative tracheitis and marked neutrophil transmigration, edema, and mucosal abscesses, but lacked hyperplasia of mucous glands.

#### Tracheobronchial lymph node (TBLN)

Mild to severe lymphoid hyperplasia, with occasional edema, was seen in all goats that were euthanized on days 2 to 14. Focal to multifocal pyogranulomas were present in all goats euthanized on days 16 and 21, and all appeared chronic (with thick fibrous capsules) on histopathology.

#### Mandibular and retropharyngeal lymph nodes

Mild to severe lymphoid hyperplasia was seen at all time points in all goats. The severity of hyperplasia was less in the mandibular lymph node compared to the retropharyngeal lymph node in all cases. Changes in these lymph nodes did not appear to correlate with the severity of hyperplasia in the TBLNs.

#### Liver

Histologically, the predominant lesion – regardless of time point – was a minimal to mild random necrosuppurative hepatitis, which was seen in six goats. Hepatic lipidosis was also noted in six goats, but was not associated with significant inflammation as the two lesions were only seen together in two goats.

#### Spleen

The only findings on days 2 and 7 were congestion, with mild lymphoid hyperplasia additionally noted in one goat. By day 14, capsular changes were present (not necessarily directly associated with abscesses), which varied slightly between individual animals, but included suppurative capsulitis with mesothelial cell hyperplasia and hypertrophy, lymphangectasia and lymphangitis, vasculitis, edema, and fibrosis (Figure 5C). Vasculitis within the parenchyma was also seen in goat 16. Abscesses had the typical appearance of the pyogranulomas that were observed in the lungs, regardless of their association with the capsule or parenchyma. Pyogranulomas were typically larger by day 21, but did not appear as active as the lesions on day 14. Mild lymphoid hyperplasia was also noted throughout the parenchyma.

#### Kidney

The apparent histologic age of renal lesions was less associated with the duration of infection than pulmonary or splenic lesions. No significant findings were seen in goats on day 2, nor in one of three goats on days 7 and 14. ‘Early’ lesions were seen in two of three goats on day 7 and one of three goats on days 14 and 21. These lesions ranged from focally hypercellular glomeruli only to glomeruli showing multifocal to global thickening due to hypertrophy of the parietal layer of Bowman’s capsule, mesangial thickening, hyperemia, minimal to mild infiltration of neutrophils, as well as variable tubulointerstitial nephritis and mineralization. The remaining three goats (one each on days 14, 16, and 21) all had ‘late’ lesions of characteristic linear abscesses extending from the cortex down into the medulla, but not involving the inner medulla or pelvis. Microscopically, the lesions appeared to be centered on the glomeruli and then spread to the tubules, culminating in tubulointerstitial nephritis with protein casts. The lesions were pyogranulomas with a core of degenerate neutrophils surrounded by a mantle of lymphocytes, histiocytes, and epithelioid macrophages (Figure 4F). Vasculitis involving both arteries and veins was seen in association with the renal lesions in two goats. Affected vessels were expanded and segmentally to circumferentially obliterated by neutrophil infiltration into the subintima and adventitia with endothelial proliferation, and/or fibrinoid necrosis (Figure 5D).

#### Adrenal gland

Acute suppuration/abscessation (without fibrosis/capsule) was noted in two goats on days 7 and 16. This spread by day 7 was the first evidence of dissemination beyond the pulmonary system and directly draining lymph nodes. Lesions were seen at the corticomedullary junction and appeared to originate from blood vessels, which had evidence of local vasculitis (Figure 5E).

#### Mesenteric lymph node

The mesenteric lymph node was typically observed to be edematous with mild to moderate lymphoid hyperplasia regardless of time point.

#### Testis

Multifocal suppurative orchitis was noted only in goat 16, which was associated with vasculitis (both arteritis and phlebitis), hemorrhage, testicular degeneration, azospermia, and giant spermatids (Figure 5F).

## Discussion

Melioidosis has a diverse clinical presentation in humans. This may be affected by individual characteristics of the infecting strain, dose, and route of infection, which is rarely known in naturally occurring cases. These unknowns make it very difficult to study the pathogenesis of melioidosis despite the large number of individual case reports and several retrospective and prospective studies. Basic pathogenesis research has been largely restricted to murine models, but additional models are necessary for a more complete understanding of the pathogenesis of infection with *B. pseudomallei*, which has a very broad host range. Additionally, new animal model systems are also important for the development and testing of novel therapeutics and vaccines.

Patients with melioidosis typically present with protean signs. Since there is no “typical” presentation, the subdivision of cases is highly variable in the human medical literature, with some papers grouping patients by duration of signs (acute vs. chronic), the presence or absence of septic shock/bacteremia, or the primary organ system involved. Pneumonia, with (76%) or without (45%) septic shock, accounts for the largest percentage of clinical presentations of naturally occurring human melioidosis [30]. In pulmonary cases, signs that are typically present include fever (50–100% of patients) [31], [32] and leukocytosis (70% of patients) [33].

In our goat model, the only consistent clinical sign of infection was fever. The degree of pyrexia appeared to parallel the increase in granulocyte count over the first seven to nine days, with peaks on day 1 and around day 7. After day 9, temperatures tended to decrease, but this decrease was not necessarily paralleled in the granulocyte count (see Figures 1 and 2). The consistency of these alterations is expected early in infection when the dominant variables of route of infection, dose, and strain are all controlled for by the nature of experimental infection. As disease progresses over time, individual host factors may become more important and account for the greater variability seen.

Other clinical signs included cough and oculonasal discharge. Nasal swabs, including those from goats with nasal discharge, were typically culture negative for *B. pseudomallei* except in the case of goat 16, where increasingly heavy discharge and growth of *B. pseudomallei* was associated with the progression towards terminal disease. Light positive growth seen on day 1 was interpreted as residual bacteria from intratracheal infection rather than dissemination or expectorated pus. This low rate of nasal shedding suggests that horizontal spread among animals via nasal secretions would be unlikely or insignificant. This appears similar to human disease where there are very few reports of person-to-person transmission [3] even though 68% of patients with evidence of pulmonary involvement are positive on sputum culture [34].

Hematogenous dissemination, the only mechanism consistent with the observed pattern of multiorgan involvement, was not directly confirmed, likely because of the transient nature of bacteremia. Positive blood cultures were only seen in goat 16, which was associated with the progression to terminal disease. The bacteremia observed in goats appears similar to the bacteremia seen in human patients, in which only 50% of patients are culture positive, and of the culture positive patients the median bacteremia is 1.1 CFU/ml [35]. Urine collected at postmortem examination was only positive in one of the four goats with renal abscessation. The goat with a positive urine culture had terminal disease at the time of collection, which could suggest that urinary shedding is a late event and/or indicative of a more severe or advanced expansion of renal abscesses into the pelvis. This could be consistent with the finding that the presence of a positive urine culture is a negative prognostic indicator in human melioidosis patients [36]. Counts ranging from 10^4^–10^6^ CFU/ml were also seen in a small number of goats used in the dose determination portion of this study (data not shown), which is comparable to counts seen in human patients [35], [36].

Thoracic radiographic findings in human melioidosis are highly variable and the incidence of specific findings also varies considerably among retrospective imaging studies of melioidosis. Radiographic signs in acutely presenting cases often have local, patchy, alveolar infiltrates, or disseminated nodular lesions, which are suggestive of metastatic (hematogenous) spread [33], [37]–[40]. Acute cases tend to have much more rapid deterioration and death [41]. The findings in subacute to chronic presentations are more variable, with some studies reporting the absence of a predominant lesion [37], while others report findings very similar to what is seen in acute cases [33]. The upper lobes appear to be more affected in melioidosis, especially in subacute/chronic cases [33], [37], [39], [40], [42], [43]. The predominant radiographic changes seen in the goats after aerosol exposure were bronchointerstitial infiltrates indicative of airway inflammation; pulmonary nodules were often initially identified in the caudal lungs and subsequently spreading to all lung lobes. This distribution was as expected with aerosol delivery (with or without secondary septicemic seeding of the lungs), but radiographically could have the same appearance as abscesses formed from the hematogenous spread of bacteria. Therefore, it is possible that the nodular appearance in human cases could be the result of inhalational and/or septicemic disease. Despite aerosol delivery of bacteria and acute fever, there was not rapid progression of disease radiographically or clinically. This is partially a function of dose as was seen in dose determination studies, where a delivered dose of 10^7^ CFU produced fulminant pulmonary disease, necessitating euthanasia 48 h PI. Additionally, the use of healthy animals, without any predisposing risk factors for melioidosis, could have limited the development of rapidly progressive disease.

Organ involvement in melioidosis can be highly variable in both humans and goats. In humans, the lungs are the most commonly affected organ, but virtually any organ can be affected [3], [30]. Data concerning organ distribution of naturally occurring melioidosis lesions seen in goats has been described in abattoir studies from Malaysia [22] and Australia [18], [44]. The pattern of organ involvement seen in the goats in these studies [18], [22], [44], previous experimental infections [27], [28], and our current model is generally similar to what is seen in human disease, with the spleen, lung, kidney, and liver being commonly affected.

One notable difference in organ involvement and lesion severity observed in goats in this study compared to mice and humans was the rarity of gross hepatic abscesses and only mild severity of microscopic changes within the liver. Previous reports of both natural and experimental melioidosis in goats have had variable findings in regards to liver lesions, with some reporting no lesions [27], [44] and others finding hepatic lesions to be quite common [18], [20], [22], [28]. This finding may be a result of strain variation, individual host/population factors, or the duration of infection. Hepatic (and splenic) abscesses are much more common in Thai patients than Australian patients [30], [33], [45], [46], which again may relate to strain variation and duration of illness.

In the current study, the lungs were the primary site of infection. Dissemination to extrapulmonary tissues appeared to be predominantly a function of time, with greater numbers of organs involved at the later time points. Dissemination to sites other than lymph nodes draining the pulmonary system was not grossly evident before day 14 with the exception of one small adrenal abscess seen in one goat on day 7. The histologic evidence suggests that the timing of dissemination was a result of the size and extent of the pulmonary pyogranuloma formation. After aerosol delivery, mucopurulent bronchopneumonia rapidly developed and soon progressed to a more severe fibrinopurulent to bronchointerstitial pneumonia with pyogranuloma formation evident radiographically, grossly, and histologically by day 7. Within the lungs, the lesions spread along interlobular septa and subpleural stroma inducing a local leukocytoclastic vasculitis where the pyogranulomas encroached upon and eventually invaded the vessel (Figure 4D). The vasculitis appeared to peak in severity around day 7. The rise in temperatures around day 7 as well as the gross appearance of the majority of the extrapulmonary lesions on day 14 or later supports the central role of the vasculitis in hematogenous dissemination of *B. pseudomallei*.

Vasculitis also appears to be the central pathologic step in the establishment and progression of pyogranuloma development in extrapulmonary organs. Vasculitis within an adrenal vessel was noted in association with abscess formation and invasion of the parenchyma. Splenic lesions in the capsule and parenchyma were also seen in association with vasculitis. The predominance of capsular pyogranulomas appears to correlate with the capsulitis observed histologically. The renal lesions suggest that the point of entry for *B. pseudomallei* is the glomerulus, with the earliest detectable lesion being hypercellular glomeruli with neutrophil infiltration. The lesions then suppurate and extend into tubules creating tubulointerstitial disease and ultimately result in the formation of pyogranulomas. The testicle was the only organ with lesions that had evidence of hemorrhage in association with the vasculitis. The significance of this finding is unclear at this time since a testicular lesion was only seen in one goat, but lends support to vasculitis being a central event in pathogenesis.

To the authors’ knowledge, vasculitis has not previously been reported as a feature of human disease or in murine models. It has been reported in goats in association with central nervous system lesions [44]. There are a limited number of papers with detailed histopathologic descriptions of human disease [12]–[15], [47], [48], none of which report vasculitis. The majority of these reports are based on acute fatal cases with lesions that are more suppurative in nature. Zones of hemorrhage, particularly surrounding pulmonary lesions are frequently reported in acute human cases [12], [13], [15], [47], [48], but have not been reported to be associated with vasculitis. A more recent paper, which also included a number of surgical biopsy samples from chronic cases in addition to acute fatal cases, specifically noted that vasculitis was not found, and only reported hemorrhage being variably present in biopsy samples [14]. The lack of vascular lesions in human cases may be a species difference or could reflect the skewed distribution of histologic samples in human cases.

Goats are generally believed to have a natural tendency towards more chronic disease and granulomatous type lesions in melioidosis [15], though disease ranging from acutely fatal to apparently self-curing lesions has been observed [25]. While there are certain notable differences in caprine and human melioidosis, the ability of goats to survive the acute stages of melioidosis, contain *B. pseudomallei* to more chronic lesions, and even potentially eliminate the organism, makes them a particularly interesting animal model of melioidosis. We have shown that melioidosis can be readily induced in goats following aerosol exposure. As expected, the extent of organ involvement seen was more variable in goats than in murine models, but was comparable to human disease and is likely to be a feature of any model with a truly heterogeneous outbred population. However, it appeared that organ involvement may become more consistent at later time points with more chronic disease in our caprine model.

The larger body size of goats allows for human-relevant clinical monitoring as well as longer-term serial evaluation of disease progression and therapy. We believe the caprine model will be a useful animal model for further investigation of the molecular pathogenesis and host response in melioidosis, as well as evaluation of preventative and therapeutic interventions.
